# Plasma lipidomic analysis of sphingolipids in patients with large artery atherosclerosis cerebrovascular disease and cerebral small vessel disease

**DOI:** 10.1042/BSR20201519

**Published:** 2020-09-18

**Authors:** Qian You, Qing Peng, Zemou Yu, Haiqiang Jin, Jing Zhang, Wei Sun, Yining Huang

**Affiliations:** Department of Neurology, Peking University First Hospital 100034, Beijing, China

**Keywords:** atherosclerosis, cerebral small vessel disease, Fabry disease, Sphingolipids

## Abstract

Background: Sphingolipids mainly consist of ceramides (Cer), sphingomyelins (SM) and glycosphingolipids. Sphingolipids are related with coronary heart disease and metabolic disease, but there’re few studies about cerebrovascular disease. The purpose was to detect sphingolipids in plasma of patients with large artery atherosclerosis (LAA) cerebrovascular disease and cerebral small vessel disease (CSVD) to explore the similarities and differences of pathogenesis of the two subtypes.

Methods: 20 patients with LAA cerebrovascular disease, 20 patients with age-related CSVD, 10 patients with Fabry disease and 14 controls were enrolled from October 2017 to January 2019. Ultra-high performance liquid chromatography-quadruple-time-of-flight mass spectrometry/mass spectrometry was used to determine sphingolipids. Univariate combined with multivariate analysis was used for comparison. Receiver operating characteristic curves were used to determine sensitivities and specificities.

Results: 276 sphingolipids were detected, including 39 Cer, 3 ceramide phosphates, 72 glycosphingolipids and 162 SM. (1) Cer (d36:3), Cer (d34:2), Cer (d38:6), Cer (d36:4) and Cer (d16:0/18:1) were increased in LAA; SM (d34:1), Cer (d34:2), Cer (d36:4), Cer (d16:0/18:1), Cer (d38:6), Cer (d36:3) and Cer (d32:0) were increased in age-related CSVD. (2) Cer (d36:4) and SM (d34:1) were increased in age-related CSVD compared with LAA. (3) Total trihexosyl ceramides were increased in Fabry group compared with control (*P*<0.05); SM (d34:1) was increased in Fabry group.

Conclusions: Ceramides are increased in both LAA and age-related CSVD, which may be related to similar risk factors and pathophysiological process of arteriosclerosis; SM is increased in both age-related CSVD and Fabry disease, suggesting that increased SM may be associated with CSVD. Glycosphingolipids, trihexosylceramides in particular, are increased in Fabry disease.

## Introduction

The 2016 Global Burden of Disease study revealed that despite a significant decline in global stroke mortality, the burden of stroke is getting heavier [1]. According to the TOAST classification, large atherosclerosis artery (LAA) cerebrovascular disease and cerebral small vessel disease (CSVD) are two common subtypes. Studies have shown that LAA accounts for 57.9% of ischemic stroke approximately, and small vessel occlusion accounts for 14.8% [2]. At present, treatment of the two remains antithrombotic therapy and control of risk factors that can be intervened. However, even with primary and secondary prevention, there are still many patients suffering from exacerbation or relapse of stroke. In addition, CSVD usually has an occult onset and progresses gradually. With pathogenic mechanism unknown and no biomarkers that can be used for early screening, current treatment is quite limited [3].

Sphingolipids mainly consist of ceramides, sphingomyelins and glycosphingolipids, varying in different lengths of fatty acyl chains and structure of unsaturated bonds [4]. Sphingolipids play important roles in multiple physiological processes such as cell proliferation, autophagy and apoptosis [5]. Experiments *in vivo* and *in vitro* have confirmed that sphingolipids can promote the development of atherosclerosis. Ceramides and sphingomyelins may affect lipoprotein metabolism, mediate oxidative stress and inflammatory response [6–9], and ceramides as well as glycosphingolipids may be related to endothelial dysfunction [10,11]. Previous clinical studies have focused more on coronary atherosclerosis [12], but less on atherosclerotic cerebrovascular disease characterized by intracranial and extracranial artery stenosis. Besides, the association between CSVD and sphingolipids is not quite clear due to lack of related studies. Therefore, our study detected the sphingolipid profiles of the two subtypes of cerebrovascular disease in order to discover sphingolipid changes and explore their similarities and differences.

Fabry disease is a rare X-linked hereditary lysosomal storage disease caused by mutations in the galactosidase (GLA) gene, which results in decreased α-GLA activity, causing glycosphingolipids, trihexosylceramides in particular, to be deposited in tissues, thus leading to multi-organ involvement [13]. Fabry disease is a hereditary CSVD, although large vessels may also be involved [14]. Sphingolipids apart from trihexo-sylceramides are less known for Fabry disease. Therefore, the present study tested the sphingolipids in plasma of patients with Fabry disease and we explored the differences of sphingolipids between age-related CSVD and the hereditary CSVD.

## Methods

### Patients

About 20 patients with LAA cerebrovascular disease, 20 patients with age-related CSVD, 10 patients with Fabry disease diagnosed at Peking University First Hospital and 20 controls were included from October 2017 to January 2019. Six controls were excluded for intracranial or extracranial artery stenosis though less than 50%. Thus, only 14 controls were enrolled eventually. The present study procedures were reviewed and approved by the Ethics Committee of Peking University First Hospital and all those enrolled had written informed consent before participation.

Inclusion criteria: LAA cerebrovascular disease: (1) Present or past history of ischemic stroke; (2) At least two types of examination including carotid artery ultrasound, transcranial Doppler (TCD), Magnetic resonance angiography (MRA), Computed tomography angiography (CTA), and Digital substraction angiography (DSA) confirmed stenosis of more than 50% in one or more intracranial or extracranial arteries; (3) The age was above 40 years. Age-related CSVD: (1) No stenosis of more than 50% in intracranial or extracranial arteries was found in carotid artery ultrasound, TCD, MRA, CTA or DSA; (2)At least one imaging manifestation including recent subcortical infarct, lacunar infarction and white matter hyperintensities (WMH) with total score of age-related white matter changes(ARWMC) >10 was present; (3)The age was above 40 years. Fabry disease: At least one of the typical symptoms including neuropathic pain, corneal involvement, and vascular keratinoma was present or having a family history. GLA mutation was definite and α-GLA enzyme activity <5% of the normal value if male [15]. Controls: (1) No history of ischemic stroke or relevant imaging manifestations on magnetic resonance imaging (MRI); (2) No stenosis in intracranial or extracranial arteries in carotid artery ultrasound, TCD, MRA, CTA or DSA; (3) No recent subcortical infarcts or lacunar infarction were present on MRI, and ARWMC score≤10.

Exclusion criteria: (1) Degenerative diseases of central nervous system such as Alzheimer’s disease and Parkinson’s disease; (2) Other non-vascular diseases which involve white matter such as inflammatory demyelinating diseases, tumors and white matter dystrophy; (3) Incomplete medical history and imaging data; (4) Other vascular diseases, such as moyamoya, vasculitis and coagulopathy; (5) Severe liver dysfunction, thyroid disease and severe infection in the past three months; (6) Cardiogenic embolism (atrial fibrillation confirmed by Holter or electrocardiogram, myocardial infarction and heart valve disease).

### Data collection

All of the clinical data needed were obtained from electronic medical records in Peking University First Hospital. Basic information including age, sex, history of hypertension, diabetes, smoking and body mass index (BMI) were recorded. Lab tests including fasting levels of total choletriglycerides (TG), total cholesterol (TC), low-density lipoprotein (LDL), high-density lipoprotein (HDL) and homocysteine (HCY) were collected. All of the enrolled performed carotid artery ultrasound, TCD, MRA or CTA to evaluate the extent of vascular stenosis and DSA was performed only if needed. Head MRI was performed to evaluate the location and severity of lesions. For those diagnosed as CSVD, different imaging manifestations, defined according to Lancet CSVD imaging standards [16], were recorded. ARWMC scores varying from 0 to 30 were also obtained [17].

### Lipidomic analysis

Reagents and materials: Peripheral blood sample collection: 2 ml venous blood was collected to the blood collection tube containing EDTA anticoagulant. Then samples were centrifuged for 5 min (4°C, 3000 r/min) within 1 h, and the precipitation was discarded before stored at −80°C.

Sample preparation: Quality control samples were prepared by mixing aliquots of all samples to be a pooled sample. (1) Took 100 μl plasma after thawed, added 300 μl isopropanol, vortexed for 30 s, sonicated for 10 min, placed at −20°C for 30 min, centrifuged for 10 min (12000 r/min, 4°C), and took 300 μl of the supernatant to fill the centrifuge tube; the quality control samples were got from mixture of equal amounts of all samples. (2) Added 200 μl isopropanol to the centrifuge tube and vortexed for 30 s; sonicated on ice for 10 min; centrifuged for 10 min (12000 r/min, 4°C), took 200 μl of the supernatant, put it in the centrifuge tube, combined the supernatant and dried; (3) Redissolved the precipitation with 200 μl isopropanol and methanol (1:1), vortexed for 30 s, sonicated for 3 min, and centrifuged for 10 min (12000 r/min, 4°C), then 150 μl of the supernatant was loaded into sample vials in liquid chromatography-mass spectrometer (LC-MS) for analysis. Quality control samples were inserted intermittently.

Lipidomic analysis: Nexera UPLC (Shimadzu, Kyoto, Japan) coupled with Q Exactive Mass spectrometer (Thermo Scientific™) was used to analyze the lipids in both heating electrospray ionization source (HESI) positive and HESI negative ion modes. Parameters of HESI were as follows: Positive ion mode: Heater Temp 300°C, Sheath Gas Flow rate 45arb, Aux Gas Flow Rate 15arb, Sweep Gas Flow Rate 1arb, spray voltage 3.5KV, Capillary Temp 320°C, S-Lens RF Level 50%, MS1 scan ranges: 120-1800. Negative ion mode: Heater Temp 300°C, Sheath Gas Flow rate 45arb, Aux Gas Flow Rate 15arb, Sweep Gas Flow Rate 1arb, spray voltage 3.1 KV, Capillary Temp 320°C, S-Lens RF Level 50%, MS1 scan ranges: 120–1800.

Waters ACQUITY UPLC BEH C18 column (100*2.1 mm,1.7 μm) were employed in both positive and negative modes. Acetonitrile and water (3/2,v/v) or isopropanol (1/9, v/v), both containing 0.1% formic acid and 10 mmol/l ammonium formate were used as mobile phases A and B, respectively. Linear gradient: 0 min, 30% B; 3 min, 30% B; 5 min, 62% B; 15 min, 82% B; 16.5 min, 99% B; 18 min, 99% B; 18.1 min, 30% B and 20 min, 30% B. The flow rate was 0.35 ml/min and column temperature was 45°C. All the samples were kept at 4°C during the analysis. The injection volume was 5 μl.

The *M/Z* of lipid molecules and lipid fragments was collected according to the following method: 10 fragment mapswere collected after each full scan (MS2 scan, HCD). MS1 has a resolution of 70000 at *M/Z* 200, and MS2 has a resolution of 17500 at *M/Z* 200.

The QCs were injected at regular intervals (every 10 samples) throughout the analytical run to provide a set of data from which repeatability can be assessed.

Lipid identification: Peaks in a raw data file of product ion scan were analyzed by Lipid-Search software (Thermo Scientific), which includes a database of more than 106 lipid species [18], using the following parameters: Precursor tolerance was set 5 ppm, product tolerance was set 8 ppm, and merge range was set 2.0 min. Each fragmented lipid was matched in the lipid database, and the fatty acid chains were determined according to the retention time. The integrated precursor extracted ion chromatogram was calculated based on the accurate mass information in order to relatively quantify the identified sphingolipids, and the area under the relative abundance curve in the integrated precursor extracted ion chromatogram was used as the sphingolipid area. We took Cer(d16:0/18:1) for an example, and its identification as well as relative quantification processes were shown in Supplementary Figure S1.

### Statistical analysis

SPSS (Version 24.0) was used for univariate analysis and SIMCA (Version 14.1) for multivariate analysis. Continuous variables were described as mean ± standard deviation (SD) (normal distribution) or median (interquartile, IQR)(non-normal distribution). Continuous variables were analyzed by one-way ANOVA or Student’s *t* test if normally distributed, and by non-parametric test if not. Categorical variables were described as frequency (percentage) and analyzed by chi-square tests. False discovery rate (FDR) was used to reduce false positive rate [19,20]. Differences were considered to be statistically significant if *P*<0.05.

Potential differential sphingolipids (*P*<0.05 before FDR correction) were selected for multivariate analysis. The unsupervised principal component analysis (PCA) was used first to observe the overall distribution. Then orthogonal partial least squares-discriminant analysis (OPLS-DA) models were established for pairwise comparison of the potential differential sphingolipids. Two hundred response permutation testing was performed to avoid model overfitting. Finally, the variable importance of projection (VIP) of each sphingolipid molecule was obtained.

Receiver operating characteristic curve (ROC) of sphingolipids whose VIP>1 and *P*<0.05 after FDR correction was performed to calculate the area under curve (AUC) and 95% confidence interval (CI) of the corresponding sphingolipids. Best cut-off value was selected using the Youden index formula, and sensitivity as well as specificity were obtained subsequently.

## Results

### Lipidomic analysis

A total of 276 sphingolipids were obtained, including 207 identified in positive ion mode, and 69 in negative ion mode. About 39 ceramides, 3 ceramide phosphates, 72 sphingolipids, and 162 sphingomyelins were successfully determined (Supplementary Table S1). Since the peak area of sphingolipid calculated is proportional to the level in plasma, it can reflect the content of the corresponding lipid in plasma. Quality control data showed the stability of the mass spectrometry platform during the experiment (Supplementary Figure S2). The other samples were not completely clustered together, indicating that there were individual differences in the groups. Since PCA did not reveal much differences in the overall distribution of sphingolipids among groups, we performed comparison of single sphingolipid molecules using supervised OPLS-DA model and univariate analysis shown as follows.

### Basic information of LAA cerebrovascular disease, age-related CSVD, control and Fabry disease groups

The 20 patients with LAA cerebrovascular disease with an average age of 62.9 ± 11.7 years, 20 patients with age-related CSVD with an average age of 62.4 ± 11.7 years, 10 patients with Fabry disease with an average age of 43.7 ± 16.2 years old, and 14 controls with an average age of 64.8 ± 13.7 years were enrolled. Age in the Fabry disease group was lower than that in the control group (*P*<0.05). There were no statistically significant differences in age, gender, hypertension, diabetes, BMI, TC, TG, LDL, HDL, HCY, ARWMC scores and the proportion of severe WMH among the LAA cerebrovascular disease, age-related CSVD and control groups, and no statistically significant differences in gender, hypertension, diabetes and regular screening lipid profile were found between Fabry disease and control groups (Table 1). In the LAA group, 40% had intracranial artery stenosis, 10% had extracranial artery stenosis, and 50% had both. In the Fabry disease group, 50% were classic, 30% were late-onset, and 20% were asymptomatic heterozygotes who were young females.

**Table 1:** Basic information of LAA cerebrovascular disease, age-related CSVD and control groups

Basic information^1^	LAA (*n*=20)	Age-related CSVD (*n*=20)	Control (*n*=14)	Fabry disease (*n*=10)	*p*^3^
Age, years	62.9 ± 11.7	62.4 ± 10.7	64.8 ± 13.7	43.7 ± 16.2	0.001
Gender					0.151
Male, *N* (%)	15 (75)	16 (80)	7 (50)	5 (50)	
Female, *N* (%)	5 (25)	4 (20)	7 (50)	5 (50)	
Hypertension, *N* (%)	17 (85)	15 (75)	8 (57)	4 (40)	0.061
Diabetes, *N* (%)	11 (55)	8 (40)	3 (21)	1 (10)	0.061
Smoking, *N* (%)	10 (50)	11 (55)	4 (29)	2 (20)	0.188
BMI, kg/m^2^	24.71 (23.24–27.60)	24.22 (21.53–27.10)	25.53 (23.33–27.75)	21.37(18.08–25.34)	0.132
TG, mmol/l	1.24 (0.96–1.58)	1.46 (0.96–1.92)	1.36 (0.98–1.84)	1.53 (0.73–2.28)	0.901
TC, mmol/l	3.68 ± 0.97	3.87 ± 1.04	4.36 ± 1.02	4.35 ± 1.18	0.187
LDL, mmol/l	2.12 ± 0.72	2.29 ± 0.85	2.60 ± 0.75	2.65 ± 1.03	0.243
HDL, mmol/l	0.93 (0.82–1.04)	0.88 (0.80–1.03)	1.11 (0.88–1.31)	1.16 (0.92–1.31)	0.091
HCY, mmol/l	12.84 (10.99–19.33)	13.26 (10.62–17.25)	13.04 (8.96–16.06)	/	0.717
Combination with severe WMH^2^, *N* (%)	5 (25%)	5 (25%)	0	/	0.106
ARWMC	5 (2–11)	7 (3–12)	3 (1-4)	/	0.095

^1^Continuous variables were described as mean±SD (normal distribution) or median (IQR)(non-normal distribution), Categorical variables were described as frequency (percentage);

^2^severe WMH was defined as ARWMC>10;

^3^Continuous variables were analyzed by one-way ANOVA if normally distributed, and by non-parametric test if not. Categorical variables were analyzed by chi-square test. *P*<0.05 was considered to be statistically different.

### Sphingolipid changes in LAA cerebrovascular disease

As is shown in Table 2, ceramides including Cer (d36:3), Cer (d34:2), Cer (d38:6), Cer (d36:4) and Cer (d16:0/18:1) were increased in LAA cerebrovascular disease than those in the control (*P*<0.05), suggesting that changes of ceramides may be present in LAA cerebrovascular disease. In order to verify their discrimination effectiveness, the five ceramides were analyzed by ROC, and their AUCs were all above 0.8. According to the Youden index formula, best cut-off values were selected. The sensitivities were above 80%, and specificities were above 85%. Therefore, ceramides were increased in LAA cerebrovascular disease.

**Table 2:** differential sphingolipids between LAA cerebrovascular disease and control

Sphingolipid^1,^^2^	Peak area		VIP	*p*^3^	AUC	95%CI	sensitivity	specificity
	LAA	Control						
Cer (d36:3)	0.015 ± 0.007	0.004 ± 0.003	1.53	<0.001	0.918 ± 0.048	0.823–1.000	80%	100%
Cer (d34:2)	0.177 (0.149–0.199)	0.076 (0.060–0.106)	1.80	<0.001	1.000 ± 0	1.000–1.000	100%	100%
Cer (d38:6)	0.026 (0.020–0.031)	0.009 (0.008–0.013)	1.53	0.002	0.939 ± 0.041	0.858–1.000	90%	85.7%
Cer (d36:4)	0.062 (0.052–0.079)	0.034 (0.032–0.038)	1.41	0.002	0.929 ± 0.044	0.842–1.000	95%	85.7%
Cer (d16:0/18:1)	0.106 ± 0.033	0.065 ± 0.013	1.31	0.002	0.889 ± 0.068	0.757–1.000	90%	92.9%

1 Described as mean±SD (normal distribution) or median (IQR) (non-normal distribution);

^2^Sphingolipids whose *P*<0.05(after FDR correction) and VIP>1;

^3^*P* value after FDR correction.

### Sphingolipid changes in age-related CSVD

As is shown in Table 3, SM (d34:1) and several ceramides including Cer (d34:2), Cer (d36:4), Cer (d16:0/18:1), Cer (d38:6), Cer (d36:3) and Cer (d32:0) were increased in age-related CSVD than those in control, suggesting there may be changes of sphingomyelin and ceramides in age-related CSVD. ROCs of the differential sphingolipids were drawn subsequently, and their AUCs were all above 0.8. Best cut-off values were selected using the Youden index formula, and their sensitivities as well as specificities were all above 85%. Besides, the sensitivities and specificities of Cer (d34:2) and Cer (d36:4) were 100%, which demonstrated fair discrimination of the two groups in this sample. Thus, Cer (d34:2), Cer (d36:4), Cer (d16:0/18:1), Cer (d38:6), Cer (d36:3), Cer (d32:0) and SM (d34:1) were increased in age-related CSVD.

**Table 3:** differential sphingolipids between age-related CSVD and control

Sphingolipid^1,^^2^	Peak area		VIP	*P*^3^	AUC	95%CI	Sensitivity	Specificity
	Age-related CSVD	Control						
Cer (d34:2)	0.175 (0.139–0.241)	0.076 (0.060–0.106)	1.16	<0.001	1.000 ± 0	1.000–1.000	100%	100%
Cer (d36:4)	0.112 (0.093–0.118)	0.034 (0.032–0.038)	1.32	<0.001	1.000 ± 0	1.000–1.000	100%	100%
Cer (d16:0/18:1)	0.137 ± 0.048	0.065 ± 0.013	1.17	<0.001	0.961 ± 0.034	0.894–1.000	95%	92.9%
Cer (d38:6)	0.028 (0.025–0.034)	0.009 (0.008–0.013)	1.15	<0.001	0.975 ± 0.022	0.931–1.000	100%	85.7%
Cer (d36:3)	0.014 ± 0.007	0.004 ± 0.003	1.12	<0.001	0.904 ± 0.054	0.797–1.000	85%	92.9%
Cer (d32:0)	0.607 ± 0.157	0.389 ± 0.071	1.14	<0.001	0.925 ± 0.044	0.838–1.000	85%	92.9%
SM (d34:1)	0.853 (0.322–1.341)	0.051 (0.020–0.120)	1.08	0.018	0.857 ± 0.071	0.717–0.997	85%	92.9%

^1^Described as mean ± SD (normal distribution) or median (IQR) (non-normal distribution);

^2^Sphingolipids whose *P*<0.05 (after FDR correction) and VIP>1;

*^3^P*-value after FDR correction.

### Differential sphingolipids between LAA cerebrovascular disease and age-related CSVD

**Figure 1:**
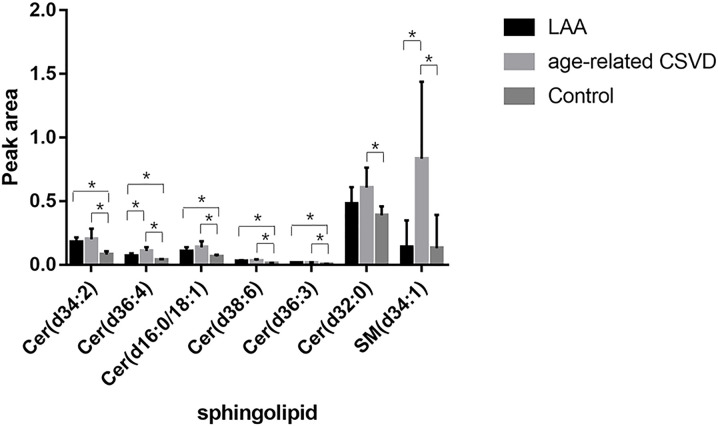
Peak area of sphingolipids among LAA cerebrovascular disease, age-related CSVD and control groups Cer (d36:3), Cer (d34:2), Cer (d38:6), Cer (d36:4) and Cer (d16:0/18:1) were increased in both LAA cerebrovascular disease and age-related CSVD groups. SM (d34:1) and Cer (d32:0) were increased in age-related CSVD. The levels of SM (d34:1) and Cer (d36:4) in the age-related CSVD were higher than those in the LAA cerebrovascular disease. *P<0.05 (after FDR correction) and VIP>1 in pairwise comparison.

The levels of SM (d34:1) and Cer (d36:4) in the age-related CSVD were higher than those in the LAA cerebrovascular disease, with AUCs greater than 0.85. Therefore, SM (d34:1) and Cer (d36:4) may be helpful to distinguish the two subtypes of cerebrovascular disease (Table 4). The results of the three groups above were summarized in Figure 1.

**Table 4:** ROC model parameters of differential sphingolipids between LAA cerebrovascular disease and age-related CSVD

Sphingolipid^1,^^2^	Peak area		VIP	*P*^3^	AUC	95%CI	Sensitivity	Specificity
	LAA	Age-related CSVD						
SM (d34:1)	0.056 (0.030–0.128)	0.853 (0.322–1.341)	1.20	0.008	0.850 ± 0.071	0.712–0.988	85%	85%
Cer (d36:4)	0.062 (0.052–0.079)	0.112 (0.093–0.118)	1.52	0.006	0.888 ± 0.055	0.780–0.995	75%	90%

^1^Described as mean±SD (normal distribution) or median (IQR)(non-normal distribution);

^2^Sphingolipids whose *P*<0.05(after FDR correction) and VIP>1;

3 *P* value after FDR correction.

### Sphingolipid changes in Fabry disease

**Figure 2:**
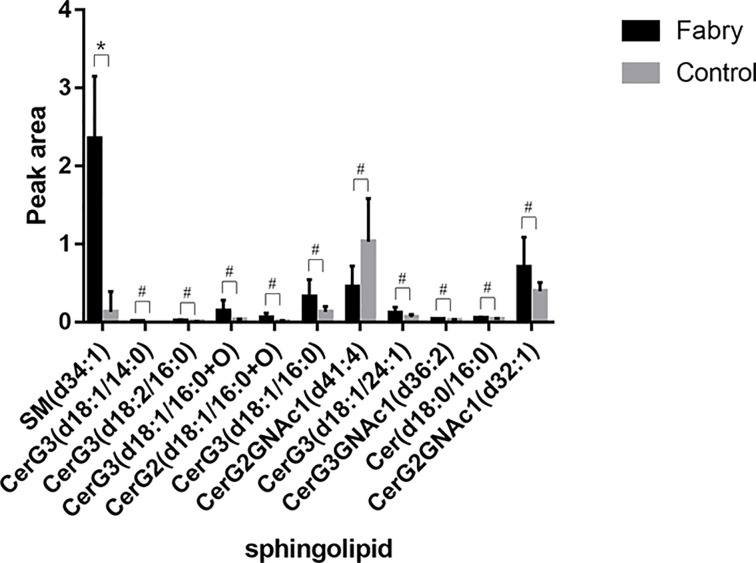
Peak area of sphingolipids between Fabry disease and control groups SM (d34:1), Cer (d18:0/16:0) and several trihexosylceramides were increased in Fabry disease group, while dihexosylceramides varied. However, only SM (d34:1) survived FDR correction. *P<0.05 (after FDR correction) and VIP>1, ^#^*P*<0.05 and VIP>1, but did not survive FDR correction.

Total level of trihexosylceramides in the Fabry disease was higher than that in the control (*P*<0.05) (Supplementary Table S3). SM (d34:1), Cer (d18:0/16:0) and several glycosphingolipids including CerG3 (d18:1/14:0), CerG3 (d18:2/16:0), CerG3 (d18:1/16:0+O), CerG3 (d18:1/16:0), CerG3 (d18:1/24:1), CerG3GNAc1 (d36:2), CerG2 (d18:1/16:0+O) as well as CerG2GNAc1 (d32:1) were increased in Fabry disease compared with control, while CerG2GNAc1 (d41:4) decreased (Figure 2). However, only SM (d34:1) survived FDR correction (*P*<0.05) (Table 5). AUC of SM (d34:1) was 1, and its sensitivity as well as specificity were both 100% by the Youden index formula, suggesting that SM (d34:1) may be increased in Fabry disease.

**Table 5:** different sphingolipids in Fabry disease versus control

Sphingolipid^1^	Peak area		VIP	*P*	*P-*value after FDR^2^
	Fabry disease	Control			
SM (d34:1)	2.216 (1.735–3.012)	0.051 (0.020–0.120)	1.68	<0.001	0.012
CerG3 (d18:1/14:0)	0.009 ± 0.008	0.002 ± 0.001	1.31	0.012	0.484
CerG3 (d18:2/16:0)	0.020 ± 0.013	0.007 ± 0.003	1.26	0.013	0.460
CerG3 (d18:1/16:0+O)	0.146 ± 0.135	0.027 ± 0.014	1.21	0.021	0.439
CerG2 (d18:1/16:0+O)	0.039 (0.016–0.107)	0.010 (0.007–0.017)	1.18	0.019	0.477
CerG3 (d18:1/16:0)	0.321 ± 0.224	0.133 ± 0.067	1.17	0.027	0.376
CerG2GNAc1 (d41:4)	0.451 ± 0.268	1.030 ± 0.555	1.11	0.003	0.405
CerG3 (d18:1/24:1)	0.119 ± 0.071	0.064 ± 0.033	1.08	0.041	0.469
CerG3GNAc1 (d36:2)	0.038 ± 0.010	0.025 ± 0.010	1.07	0.005	0.498
Cer (d18:0/16:0)	0.052 ± 0.012	0.039 ± 0.010	1.06	0.008	0.555
CerG2GNAc1 (d32:1)	0.702 ± 0.386	0.396 ± 0.112	1.05	0.035	0.459

^1^Described as mean ± SD (normal distribution) or median (IQR) (non-normal distribution);

^2^*P*<0.05 was considered to be statistically different.

## Discussion

### LAA cerebrovascular disease

Ceramides are backbone components of the family of sphingolipids. In our study, we found there were changes in the levels of ceramides in LAA cerebrovascular disease. There have been few previous studies on LAA cerebrovascular disease and ceramides. Previous studies have shown that ceramides, as second messenger molecules, participate in inflammatory pathways and oxidative stress [7,21]. In addition, ceramides are one of the structural components of LDL [22] that participates multiple pathological processes in atherosclerosis. Besides, our team also confirmed inhibition of ceramide synthesis could reduce lipid deposition and inflammatory response in vascular wall of ApoE-/-mice so as to delay progression of atherosclerosis [23].

We noticed that the length and saturation of fatty acyl chains in the ceramides whose levels were higher than control varied. Six kinds of ceramide synthetase (CerS) are involved in the *de novo* synthesis of ceramides, which are related to tissue distribution and different fatty acyl chain lengths varying from Cer14:0 to Cer30:0 [24,25]. However, the physiological characteristics of ceramides with different fatty acyl chains remain largely unknown. Studies have found that increase of long-chain ceramides leads to β-oxidation impairment, which is related to diabetes and insulin resistance vice versa [26–28]. In our study, the level of Cer(d16:0/18:1) in the LAA cerebrovascular disease group was increased, consistent with previous reports. However, fatty acyl chain of the other four molecules failed to get identified.

In a word, our study found ceramide molecules were increased in LAA cerebrovascular disease. Most patients in the LAA cerebrovascular disease group had been treated with statins to reduce cholesterol and LDL, but they still suffered from stroke.Animal experiments of our team showed that myriocin’s inhibitory effect on atherosclerosis was independent of LDL [23]. Therefore, sphingolipids represented by ceramides may have a pathogenic effect on LAA cerebrovascular disease and may be potential therapeutic targets, especially for those with poor therapeutic effects with statins.

### Age-related CSVD

In the present study, we found several ceramides and SM (d34:1) were increased in age-related CSVD. Ceramides may cause endothelial dysfunction and increase the permeability of the blood–brain barrier by promoting oxidative stress, inflammation and reducing the production of nitric oxide [29–31], thus leading to CSVD [32]. Ceramides and sphingomyelins are components of myelin sheath in white matter, playing important roles in maintaining normal function of the myelin sheath [33]. Demyelination is a common pathogenic manifestation in WMH [34], so we speculated that increase of ceramides as well as sphingomyelins may be related to WMH, consistent with previous research results [35].

CSVD often hides its onset, gradually progresses, and seriously affects the life quality of patients. However, with the pathogenic mechanism unclear, reliable biomarkers and treatment are limited. We proposed the several ceramides that were increased in age-related CSVD may serve as potential biomarkers of CSVD in the future and provide clues for further investigation of pathogenesis of CSVD.

### Comparison of sphingolipids between LAA cerebrovascular disease and age-related CSVD

Since there are similarities as well as differences of pathogenesis of LAA cerebrovascular disease and age-related CSVD, our study also compared their sphingolipid profiles. It turned out that levels of Cer (d36:4) and SM (d34:1) in age-related CSVD were significantly higher than those in LAA cerebrovascular disease. We supposed they may help to distinguish the two subtypes of cerebrovascular disease.

We noticed that there were several ceramides in common in the LAA cerebrovascular disease and age-related CSVD when they were compared with control, probably due to enrollment of patients with acute stroke. Studies have found several ceramides were increased after stroke [36,37]. However, there were no statistically significant differences in the proportion of patients with acute stroke between the LAA cerebrovascular disease and age-related CSVD groups.

As for differences of the sphingolipid profiles between the two, metabolism disturbance of sphingomyelin was more evident in age-related CSVD, while glycosphingolipids seemed to be increased in LAA cerebrovascular disease (Supplementary Table S2), providing us with potential clues for their distinct pathogenic mechanisms and helping us with differential diagnosis. Larger samples for verification and more research of related mechanisms will be needed in the future.

### Fabry disease

Peak area of SM (d34:1) in the Fabry disease was found to be higher than that of the control, suggesting that there may be sphingomyelin metabolism abnormality in Fabry disease. Fabry disease, a kind of hereditary CSVD, could manifest as WMH, which we speculated may partially explain the increased level of sphingomyelin. Besides, Brogden et al. extracted skin fibroblasts from Fabry patients and found that sphingomyelins in the cell membrane of non-lipid raft regions were significantly increased, and decreased to normal after treatment of glycosphingolipid inhibitors [38]. However, more related studies are needed to confirm the correlation between sphingomyelin and Fabry disease.

In addition, our study showed that total level of trihexosylceramides and several trihexosylceramides were higher in Fabry disease compared with control, and the degree varies for different molecules. They did not survive FDR correction probably due to limited samples. It has been shown different trihexosylceramides are deposited in different organs, leading to different pathophysiological changes. Levels of dihexosylceramides varied in our study, consistent with previous reports [39]. More research are still needed in the future to help us understand properties of different glycosphingolipids and their mechanism of regulation.

Furthermore, there were some differences between Fabry disease and age-related CSVD: in Fabry disease, it was dominated by glycosphingolipid changes, while in age-related CSVD ceramide changes were more prominent. Our study showed there were not statistically significant differences of ceramides in Fabry disease compared with control, consistent with previous reports [39,40]. It is currently believed that the vascular involvement of Fabry disease is related to the deposition of glycosphingolipids in endothelial cells and smooth muscle cells, probably due to induction of oxidative stress [41,42]. There are not significant differences on the severity or spatial distribution of lacunar infarction and WMH in Fabry disease and age-related CSVD [43,44], making it difficult to distinguish them from imaging manifestations. Therefore, detection of ceramides and glycosphingolipids may help with differential diagnosis of age-related CSVD and Fabry disease.

There were several limitations in our study. Lipidomics is often used as a tool for lipid biomarker screening and drug target exploration. However, determination and quantification of sphingolipid profiles are difficult, considering their diversity and structural complexity. Therefore, the sample size is limited. The sphingolipid changes of LAA cerebrovascular disease, age-related CSVD and Fabry disease found in the present study still need to be verified by large-scale trials. In the present study, inclusion criteria of control was non-stenosis of intracranial and extracranial large vessels. However, atherosclerosis is a systemic vascular disease, and the control did not undergo relevant examination of other vessels such as coronary artery and arteries of the extremities, though symptoms of chest pain, cold extremities and intermittent claudication were not present. In addition, we failed to identify fatty acyl chain lengths and saturation bonds in some of the sphingolipids. It remains unclear whether sphingolipids with different fatty acyl chain lengths and saturation bonds participate in different physiological processes, which needs further exploration. Last but not the least, the present study focused on ischemic CSVD, and cerebral microbleeds were not involved. However, differences between the pathogenesis of ischemic CSVD and that of hemorrhagic CSVD remain unknown. There is not related research on whether lipids play a role, which is worth further exploration.

## Supplementary Material

Supplementary Figures S1-S2 and Tables S1-S3

## Abbreviations

ARWMC: age-related white matter changes
AUC: area under curve
BMI: body mass index
Cer: ceramides
CerS: ceramide synthetase
CI: confidence interval
CSVD: cerebral small vessel disease
CTA: computed tomography angiography
DSA: digital substraction angiography
eNOS: endothelial nitric oxide synthase
FDR: false discovery rate
GLA: galactosidase
HCY: homocysteine
HDL: high-density lipoprotein
IQR: interquartile
LAA: large artery atherosclerosis
LC-MS: liquid chromatography-mass spectrometer
LDL: low-density lipoprotein
MRA: magnetic resonance angiography
MRI: magnetic resonance imaging
NO: nitric oxide
OPLS-DA: orthogonal partial least squares-discriminant analysis
PCA: principal component analysis
ROC: receiver operating characteristic curve
SD: standard deviation
SM: sphingomyelins
TC: total cholesterol
TCD: transcranial Doppler
TG: choletriglycerides
VIP: variable importance of projection
WMH: white matter hyperintensities
