# Supplementary material for: Plasma lipidomic analysis of sphingolipids in patients with large artery atherosclerosis cerebrovascular disease and cerebral small vessel disease
Source: Biosci Rep. 2020 Sep 18;40(9):BSR20201519. doi: 10.1042/BSR20201519 (PMC7502657; doi:10.1042/BSR20201519)
Supplement: Supplementary Figures S1-S2 and Tables S1-S3 [file BSR-2020-1519_supp.pdf]

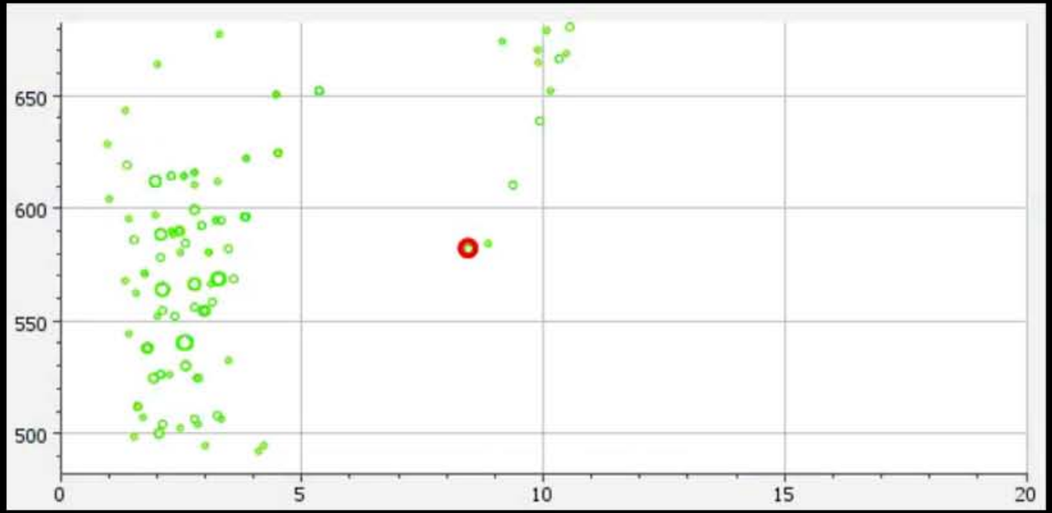

650
600
550
500
0
5
10
15
20

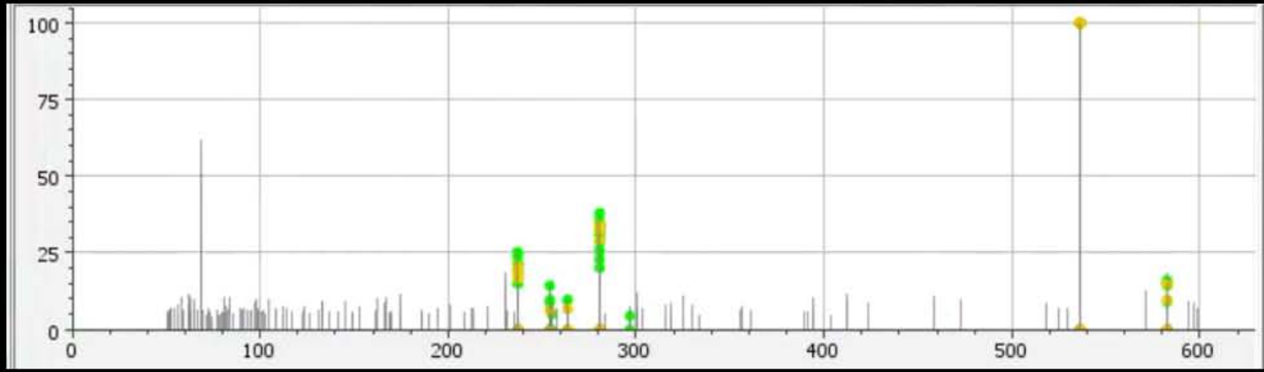

100
75
50
25
0
0
100
200
300
400
500
600

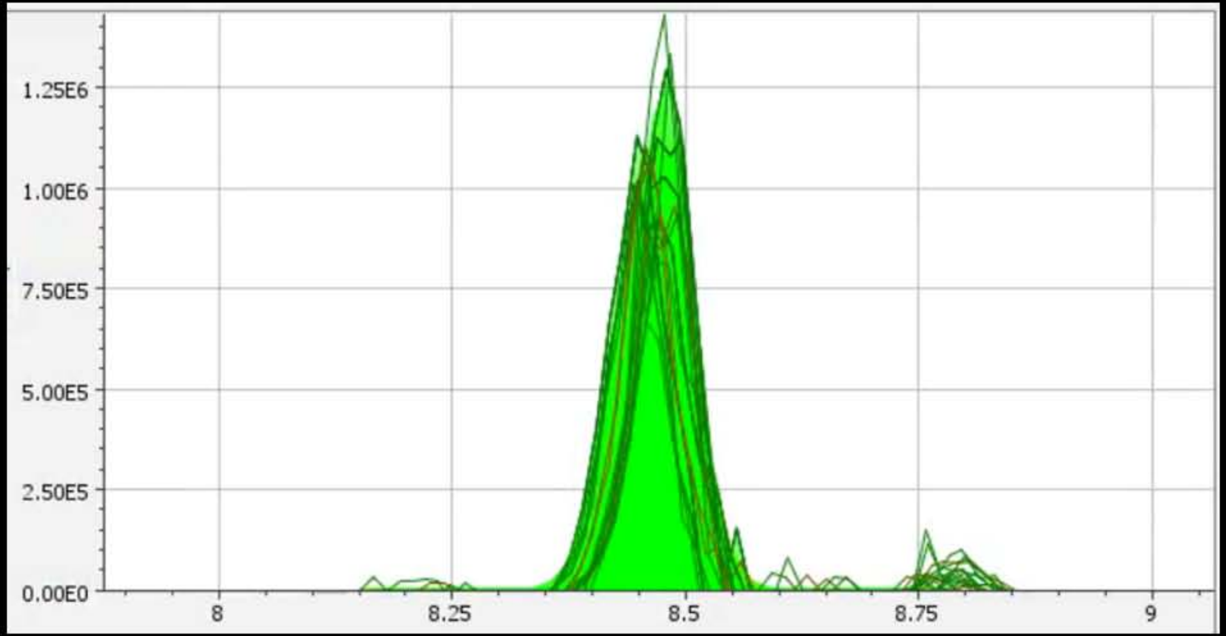

1.25E6
1.00E6
7.50E5
5.00E5
2.50E5
0.00E0
8
8.25
8.5
8.75
9

Supplemental Figure 1. Identification and relative quantification of Cer(d16:0/18:1). Cer(d16:0/18:1)had m/z of 538.519 and a retention time of 8.0035min. Its identification and relative quantification were shown from LipidSearch software. The distribution of mass to charge ratio (m/z) detected from lipid extractsduring retention time 0 to 20 minin MS datawas shown, and the one with red circle in A was identified as Cer(d16:0/18:1) according to the product ion spectra shown in B. The relative quantification of Cer (d16:0/18:1) was computed by its precursor ions from full-scan MS and integrated extracted ion chromatograms.

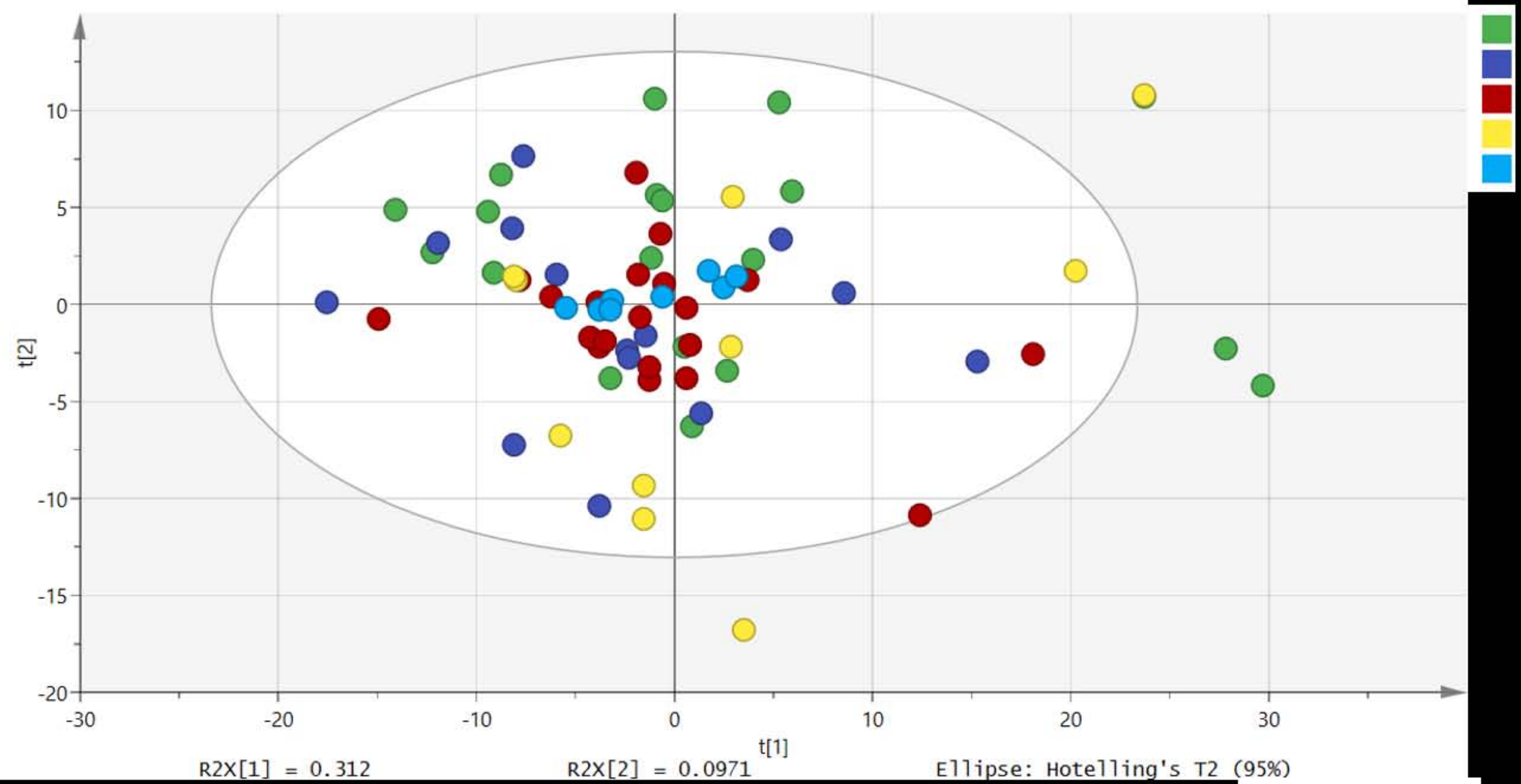

10
5
0
-5
-10
-15
-20
t[2]
-30
-20
-10
0
10
20
30
t[1]
R2X[1] = 0.312
R2X[2] = 0.0971
Ellipse: Hotelling's T2 (95%)

Supplemental Figure 2. Principal component analysis model of sphingolipids. Principle component analysis of LAA cerebrovascular disease, age-related CSVD, Fabry disease and control groups as well as quality control group (R^2X=0.682, Q^2 =0.43).The quality control samples were closely clustered, indicating good stability.

Supplemental Table 1. **Sphingolipids detected in lipidomic analysis**

Sphingolipid	m/z	Classification
Cer(d18:1/26:4)	670.613	Cer
Cer(d18:1/32:5)	752.692	Cer
Cer(d15:0/26:1)	634.614	Cer
Cer(d42:1+O)	664.625	Cer
Cer(d44:4)	672.629	Cer
Cer(d18:1/22:0)	620.599	Cer
Cer(d18:1/24:1)	648.629	Cer
Cer(d30:0)	484.472	Cer
Cer(d36:3)	562.519	Cer
Cer(d24:0/17:0+O)	654.639	Cer
Cer(d18:2/24:0)	646.614	Cer
Cer(d16:1)	284.223	Cer
Cer(d42:4)	644.598	Cer
Cer(d18:0/24:1)	650.645	Cer
Cer(d18:0/20:0)	596.598	Cer
Cer(d18:1/24:0)	648.63	Cer
Cer(d18:1/18:0)	566.551	Cer
Cer(d22:0/18:0+O)	640.624	Cer
Cer(d18:0/16:0)	540.535	Cer
Cer(d18:1/24:2)	646.613	Cer
Cer(d18:0/24:0)	652.66	Cer
Cer(d17:1/24:0)	636.629	Cer
Cer(d20:1/24:1)	676.66	Cer
Cer(d18:0/22:0)	624.629	Cer
Cer(d18:0/18:0)	568.566	Cer
Cer(d17:0/24:0)	638.645	Cer
Cer(d20:1/24:0)	678.676	Cer
Cer(d16:0/18:1)	538.519	Cer
Cer(d17:1/22:0)	608.598	Cer
Cer(d18:1/16:0)	538.519	Cer
Cer(d18:1/20:0)	594.582	Cer
Cer(d18:2/22:0)	620.598	Cer

Cer(d24:0/18:0+O)	668.655	Cer
Cer(d38:6)	584.504	Cer
Cer(d18:0)	316.285	Cer
Cer(d32:0)	512.504	Cer
Cer(d34:0)	540.535	Cer
Cer(d34:2)	536.504	Cer
Cer(d36:4)	560.504	Cer
CerG1(d18:1/20:0)	756.635	CerG1
CerG1(d18:2/22:2)	778.619	CerG1
CerG1(d40:1)	782.652	CerG1
CerG1(d34:0+2O)	734.578	CerG1
CerG1(d18:2/22:0)	782.65	CerG1
CerG1(d18:1/24:2)	808.666	CerG1
CerG1(d22:0/20:5)	804.635	CerG1
CerG1(d42:1)	834.679	CerG1
CerG1(d18:1/16:0+O)	716.567	CerG1
CerG1(d18:1/26:5)	830.65	CerG1
CerG1(d18:1/24:1)	810.682	CerG1
CerG1(d18:1/16:0)	700.572	CerG1
CerG1(d18:1/22:0)	784.666	CerG1
CerG1(d17:1/24:0)	798.682	CerG1
CerG1(d18:1/24:0)	812.697	CerG1
CerG1(d18:0/16:0+2O)	734.578	CerG1
CerG1(d43:4)	820.666	CerG1
CerG1(d42:4)	806.65	CerG1
CerG1(d44:4)	834.682	CerG1
CerG2(d18:1/16:0+O)	878.62	CerG2
CerG2(d18:1/22:0)	946.719	CerG2
CerG2(d29:0)	792.548	CerG2
CerG2(d34:4)	854.564	CerG2
CerG2(d31:1)	818.564	CerG2
CerG2(d18:1/24:2)	970.719	CerG2
CerG2(d36:5)	882.594	CerG2
CerG2(d44:5)	994.719	CerG2
CerG2(d18:1/26:3)	996.735	CerG2

CerG2(d34:1)	860.61	CerG2
CerG2(d18:2/16:0)	860.609	CerG2
CerG2(d18:1/24:1)	972.735	CerG2
CerG2(d18:1/24:0)	974.75	CerG2
CerG2(d16:1/16:0)	834.594	CerG2
CerG2(d18:1/16:0)	862.625	CerG2
CerG2GNAc1(d43:4)	591.388	CerG2GNAc1
CerG2GNAc1(d41:4)	577.373	CerG2GNAc1
CerG2GNAc1(d34:2)	1061.67	CerG2GNAc1
CerG2GNAc1(d32:1)	1035.66	CerG2GNAc1
CerG2GNAc1(d36:3)	1087.69	CerG2GNAc1
CerG2GNAc1(d36:1)	1091.72	CerG2GNAc1
CerG3(d18:1/18:0)	1052.71	CerG3
CerG3(d18:1/16:0+O)	1040.67	CerG3
CerG3(d18:1/14:0)	996.647	CerG3
CerG3(d18:1/22:0)	1108.77	CerG3
CerG3(d18:2/16:0)	1022.66	CerG3
CerG3(d18:1/24:0)	1136.8	CerG3
CerG3(d18:1/24:1)	1134.79	CerG3
CerG3(d18:1/16:0)	1024.68	CerG3
CerG3GNAc1(d34:1)	1225.74	CerG3GNAc1
CerG3GNAc1(d36:2)	1253.77	CerG3GNAc1
CerG3GNAc1(d42:2)	1337.87	CerG3GNAc1
CerG3GNAc2(d34:2+O)	1444.82	CerG3GNAc2
CerP(d18:1/16:0)	618.486	CerP
CerP(d22:0/26:6)	804.627	CerP
CerP(d47:7)	786.581	CerP
GD1a(d34:1)	903.463	GD1a
GD1a(d36:1)	917.479	GD1a
GD3(d16:0/18:1)	720.897	GD3
GM1(d34:1)	1516.84	GM1
GM3(d39:1)	1221.78	GM3
GM3(d42:1+O)	1279.83	GM3
GM3(d36:2)	1179.74	GM3
GM3(d41:1)	1251.83	GM3

GM3(d36:1)	1181.75	GM3
GM3(d32:1)	1125.69	GM3
GM3(d42:3)	1259.8	GM3
GM3(d42:1)	1263.83	GM3
GM3(d40:1)	1237.81	GM3
GM3(d34:2)	1151.7	GM3
GM3(d40:1+O)	1251.79	GM3
GM3(d34:1+O)	1169.72	GM3
GM3(d42:2+O)	1277.81	GM3
GM3(d38:1)	1207.77	GM3
GM3(d42:2)	1263.83	GM3
GM3(d34:1)	1151.71	GM3
phSM(d40:4)	841.608	phSM
phSM(d20:1)	523.351	phSM
phSM(d38:3)	771.601	phSM
phSM(d40:1)	803.664	phSM
phSM(d40:7)	791.57	phSM
phSM(d41:2)	815.664	phSM
phSM(d42:7)	819.601	phSM
phSM(d46:7)	875.664	phSM
phSM(d46:8)	873.648	phSM
phSM(d20:1/16:0)	791.592	phSM
phSM(d33:1)	705.554	phSM
phSM(d34:2)	717.554	phSM
phSM(d32:1)	735.529	phSM
phSM(d42:1)	875.686	phSM
phSM(d36:1)	747.601	phSM
phSM(d36:3)	743.57	phSM
phSM(d34:1)	719.57	phSM
phSM(d44:4)	853.679	phSM
phSM(d22:0/20:3)	871.655	phSM
phSM(d42:0)	833.711	phSM
phSM(d36:4)	741.554	phSM
phSM(d22:0/20:2)	873.67	phSM
phSM(d40:0)	805.679	phSM

phSM(d18:0/16:0)	765.576	phSM
phSM(d34:0)	721.585	phSM
phSM(d18:0/16:1)	763.561	phSM
SM(d16:1/20:4)	767.534	SM
SM(d17:1/20:0)	767.604	SM
SM(d18:1/19:0)	745.622	SM
SM(d18:2/21:0)	793.619	SM
SM(d18:2/21:1)	769.622	SM
SM(d18:1/23:3)	795.637	SM
SM(d18:2/24:2)	831.635	SM
SM(d18:1/24:4)	829.619	SM
SM(d18:2/24:4)	827.604	SM
SM(d33:0)	735.566	SM
SM(d34:1)	747.566	SM
SM(d40:1)	831.66	SM
SM(d42:2)	857.675	SM
SM(d34:2)	723.541	SM
SM(d35:2)	715.575	SM
SM(d35:5)	709.528	SM
SM(d37:3)	741.591	SM
SM(d38:2)	779.604	SM
SM(d40:4)	781.622	SM
SM(d42:7)	803.606	SM
SM(d47:8)	871.669	SM
SM(d20:0/22:6)	849.613	SM
SM(d18:2/23:3)	793.622	SM
SM(d40:6)	777.591	SM
SM(d43:8)	815.606	SM
SM(d18:2/20:1)	755.606	SM
SM(d18:1/20:3)	753.591	SM
SM(d40:0)	833.675	SM
SM(d39:8)	759.544	SM
SM(d40:5)	779.606	SM
SM(d16:1/24:4)	779.606	SM
SM(d18:2/22:1)	783.637	SM

SM(d41:0)	847.691	SM
SM(d37:1)	745.622	SM
SM(d39:4)	767.606	SM
SM(d42:0)	817.716	SM
SM(d44:4)	837.684	SM
SM(d18:1/26:1)	863.698	SM
SM(d44:7)	831.637	SM
SM(d46:8)	857.653	SM
SM(d35:0)	763.597	SM
SM(d40:3)	783.637	SM
SM(d18:1/21:1)	771.637	SM
SM(d17:1/24:1)	821.651	SM
SM(d44:1)	865.713	SM
SM(d39:0)	819.66	SM
SM(d31:1)	683.51	SM
SM(d18:1/23:0)	823.666	SM
SM(d18:1/24:5)	805.622	SM
SM(d18:2/27:4)	847.669	SM
SM(d38:0)	761.653	SM
SM(d18:2/16:0)	701.559	SM
SM(d34:0)	705.591	SM
SM(d17:1/24:2)	819.635	SM
SM(d46:4)	865.716	SM
SM(d18:1/20:0)	781.619	SM
SM(d18:2/22:3)	779.606	SM
SM(d17:1/22:0)	795.635	SM
SM(d43:3)	825.684	SM
SM(d18:1/18:0)	753.588	SM
SM(d30:2)	645.497	SM
SM(d18:2/17:0)	737.557	SM
SM(d33:4)	683.512	SM
SM(d44:8)	829.622	SM
SM(d18:2/24:1)	811.669	SM
SM(d18:2/24:0)	835.666	SM
SM(d18:2/26:4)	833.653	SM

SM(d20:0/20:4)	825.613	SM
SM(d16:1/18:3)	697.528	SM
SM(d30:1)	669.494	SM
SM(d36:0)	755.604	SM
SM(d37:5)	737.559	SM
SM(d18:1/24:0)	837.682	SM
SM(d18:0/18:3)	727.575	SM
SM(d18:2/14:0)	695.51	SM
SM(d17:1/18:3)	711.544	SM
SM(d17:1/16:0)	711.541	SM
SM(d32:4)	669.497	SM
SM(d16:1/16:0)	697.525	SM
SM(d44:3)	839.7	SM
SM(d18:2/22:0)	807.635	SM
SM(d38:3)	799.597	SM
SM(d38:4)	753.591	SM
SM(d45:5)	849.684	SM
SM(d43:2)	849.682	SM
SM(d18:1/20:5)	749.559	SM
SM(d18:2/26:5)	831.637	SM
SM(d20:1/26:4)	863.7	SM
SM(d18:2/18:3)	723.544	SM
SM(d18:0/20:0)	783.635	SM
SM(d18:1/21:0)	773.653	SM
SM(d45:4)	851.7	SM
SM(d17:1/20:3)	739.575	SM
SM(d33:2)	731.534	SM
SM(d43:1)	851.698	SM
SM(d18:1/17:0)	739.572	SM
SM(d40:7)	775.575	SM
SM(d17:1/26:5)	819.637	SM
SM(d22:0/16:0)	805.644	SM
SM(d18:0/22:0)	811.666	SM
SM(d18:1/26:3)	837.684	SM
SM(d18:2/18:0)	729.591	SM

SM(d18:1/25:3)	823.669	SM
SM(d34:5)	695.512	SM
SM(d18:0/16:0)	727.572	SM
SM(d18:2/20:0)	757.622	SM
SM(d17:1/26:4)	821.653	SM
SM(d18:1/26:4)	835.669	SM
SM(d22:1/18:1)	829.644	SM
SM(d17:1/24:3)	795.637	SM
SM(d16:1/26:4)	807.637	SM
SM(d25:0/18:2)	871.691	SM
SM(d42:4)	809.653	SM
SM(d20:0/18:1)	803.628	SM
SM(d18:1/22:2)	783.637	SM
SM(d42:5)	851.628	SM
SM(d37:2)	787.597	SM
SM(d18:2/19:0)	743.606	SM
SM(d22:0/20:3)	855.66	SM
SM(d18:1/26:0)	843.731	SM
SM(d32:0)	721.55	SM
SM(d39:2)	771.637	SM
SM(d22:1/20:1)	857.675	SM
SM(d25:0/18:1)	873.707	SM
SM(d22:2/17:0)	815.628	SM
SM(d20:0/16:0)	777.613	SM
SM(d16:1/14:0)	647.512	SM
SM(d17:1/14:0)	661.528	SM
SM(d20:1/15:0)	761.581	SM
SM(d22:2/19:1)	841.644	SM
SM(d22:2/22:1)	883.691	SM
SM(d18:1/25:0)	829.716	SM
SM(d18:1/26:2)	839.7	SM
SM(d41:1)	845.675	SM
SM(d44:2)	885.707	SM
SM(d41:3)	797.653	SM

Supplemental Table 2. **Total mount of different subclasses of sphingolipids in Age-related CSVD and LAA groups**

Classification[a]		Age-related CSVD	LAA	p_b
Cer[c]		10.78±1.71	10.70±1.77	0.881
CerP		0.42±0.09	0.41±0.14	0.757
SM		631.21 (588.86-679.90)	593.80(494.07-657.02)	0.194
Glycosphingolipids	Total	22.61±3.43	25.67±4.37	0.018
	CerG1	5.27±1.98	5.77±2.13	0.387
	CerG2	10.60 (10.04-11.83)	11.81 (10.46-14.80)	0.023
	CerG3	0.45 (0.38-0.53)	0.46 (0.36-0.62)	0.829
	GM	5.98 (5.31-7.14)	5.97 (5.17-6.72)	0.871

a. described as mean±SD (normal distribution) or median (IQR)(non-normal distribution);

b. It was analyzed by student t test if normally distributed, and by non-parametric test if not. $p<0.05$ was considered to be statistically different;

c. Cer-ceramide, CerP-ceramide phosphate, SM-sphingomyelin, CerG1-monohexosylceramide, CerG2-dihexosylceramide, CerG3-trihexosylceramide, GM-ganglioside.

Supplemental Table 3. **Total mount of different subclasses of sphingolipids in Fabry disease and control**

Classification[a]		Fabry disease	Control	p_b
Cer[c]		10.01±1.80	9.86±1.40	0.827
CerP		0.39 (0.33-0.39)	0.43(0.27-0.46)	0.770
SM		664.74±113.31	623.67±116.55	0.399
Glycosphingolipids	Total	25.10±2.72	23.28±4.07	0.233
	CerG1	5.91±1.87	5.35±1.79	0.464
	CerG2	11.66±3.39	11.09±1.70	0.589
	CerG3	1.01±0.63	0.52±0.25	0.037
	GM	6.51±0.84	6.33±1.26	0.686

a. described as mean±SD (normal distribution) or median (IQR)(non-normal distribution);

b. It was analyzed by student t test if normally distributed, and by non-parametric test if not. $p<0.05$ was considered to be statistically different.

c.. Cer-ceramide, CerP-ceramide phosphate, SM-sphingomyelin, CerG1-monohexosylceramide, CerG2-dihexosylceramide, CerG3-trihexosylceramide, GM-ganglioside.
